# Hypoxia Inhibits Osteogenesis in Human Mesenchymal Stem Cells through Direct Regulation of RUNX2 by TWIST

**DOI:** 10.1371/journal.pone.0023965

**Published:** 2011-09-09

**Authors:** Der-Chih Yang, Muh-Hwa Yang, Chih-Chien Tsai, Tung-Fu Huang, Yau-Hung Chen, Shih-Chieh Hung

**Affiliations:** 1 Institute of Clinical Medicine, National Yang-Ming University, Taipei, Taiwan; 2 Pharmacology, National Yang-Ming University, Taipei, Taiwan; 3 Department of Surgery, National Yang-Ming University, Taipei, Taiwan; 4 Department of Chemistry, Tamkang University, Taipei, Taiwan; 5 Department of Medical Research and Education, Veterans General Hospital-Taipei, Taipei, Taiwan; 6 Orthopaedics and Traumatology, Veterans General Hospital-Taipei, Taipei, Taiwan; 7 Hematology-Oncology, Veterans General Hospital-Taipei, Taipei, Taiwan; Brigham and Women's Hospital, United States of America

## Abstract

**Background:**

Bone loss induced by hypoxia is associated with various pathophysiological conditions, however, little is known about the effects of hypoxia and related signaling pathways on osteoblast differentiation and bone formation. Because bone marrow-derived mesenchymal stem cells (MSCs) survive under hypoxic conditions and readily differentiate into osteoblasts by standard induction protocols, they are a good *in vitro* model to study the effects of hypoxia on osteoblast differentiation.

**Methodology/Principle Findings:**

Using human MSCs, we discovered TWIST, a downstream target of HIF-1α, was induced under hypoxia and acted as a transcription repressor of RUNX2 through binding to the E-box located on the promoter of *type 1 RUNX2*. Suppression of *type 1 RUNX2* by TWIST under hypoxia further inhibited the expression of *BMP2*, *type 2 RUNX2* and downstream targets of *RUNX2* in MSCs.

**Conclusions/Significance:**

Our findings point to the important role of hypoxia-mediated signalling in osteogenic differentiation in MSCs through direct regulation of RUNX2 by TWIST, and provide a method for modifying MSC osteogenesis upon application of these cells in fracture healing and bone reconstruction.

## Introduction

Bone loss induced by hypoxia is associated with various pathophysiological conditions such as ischemia [1], vascular diseases [2], [3], and osteolytic bone metastases [4]. Although, hypoxia was reported to control osteoclast size and numbers [5], however, little is known about the effects of hypoxia on osteoblast differentiation and bone formation.

RUNX2 (also known as CBFA1) is a master regulator of skeletogenesis and its expression is required for the expression of several downstream genes that are important for osteoblast differentiation and maturation [6], [7]. The major isoforms of *RUNX2* involved in osteogenesis are *type1* (*T1*) and *type2* (*T2*) *RUNX2*. *T1 RUNX2* is regulated by a proximal promoter *P2* and the translation begins from the exon2 amino acid sequences (MRIPVD); whereas *T2 RUNX2* is regulated by a distal promoter *P1* and translation begins from the exon1 amino acid sequences (MASNSL). *T1 RUNX2*, chiefly expresses in early precursors of osteoblasts and chondrocytes [8], and is upregulated by growth factors such as fibroblast growth factor 2 (FGF2) to stimulate the production of bone morphogenetic protein2 (BMP2). This then enhances the transcription of *T2 RUNX2* to stimulate osteoblast differentiation and maturation [9].

The transcriptional response to hypoxia is mediated by the hypoxia-inducible transcription factor (HIF-1), a heterodimer consisting of the constitutively expressed aryl hydrocarbon receptor nuclear translocator (ARNT) and the hypoxic response factor HIF-1α. HIF-1α is regulated by the cellular O_2_ concentration and determines the transcriptional activity of HIF-1 [10]. Twist, a basic helix-loop-helix (bHLH) transcription factor, has been known to promote tumor metastasis by inducing epithelial-mesenchymal transition (EMT) [11]. Recently, Twist is known as one of the downstream targets of HIF-1α and the HIF-Twist pathway is involved in hypoxia-induced increase of metastasis in head and neck cancer [12] and hypoxia-mediated inhibition of replicative senescence and loss of stemness occurred upon expansion of adult stem cells [13].

Human multipotent stromal cells or mesenchymal stem cells (MSCs), capable of self renewal and differentiating into various mesenchymal tissues [14], have emerged as a promising tool for clinical applications in, for example, cell-based therapy for osteogenesis imperfecta [15] and tissue engineering in cartilage and bone [16]. MSCs reside in bone barrow and are easily isolated by plastic-adherence. They are the in vivo precursors of osteoblasts and are readily induced to undergo osteoblastic differentiation by standard induction protocols. Therefore they are a good non-cancerous model to study osteogenic differentiation and bone formation [12], [17].

Because MSCs isolated from bone marrow, which is hypoxic in nature (1–7% O_2_), survive under hypoxia [18], we used MSCs as the cell model to study the underlying mechanism involved in hypoxia-mediated inhibition of osteogenesis. Since the TWIST levels are increased in MSCs cultured under hypoxic conditions, remain high in freshly purified MSCs, and are downregulated following ex vivo expansion, we specifically focused on the role of Twist in modulating of osteogenesis of MSCs under hypoxic conditions [19], [20]. Our findings provide evidence that hypoxia inhibits MSC osteogenesis through direct downregulation of RUNX2 by TWIST.

## Results

### Hypoxia inhibits osteogenic differentiation by MSCs

**Figure 1:**
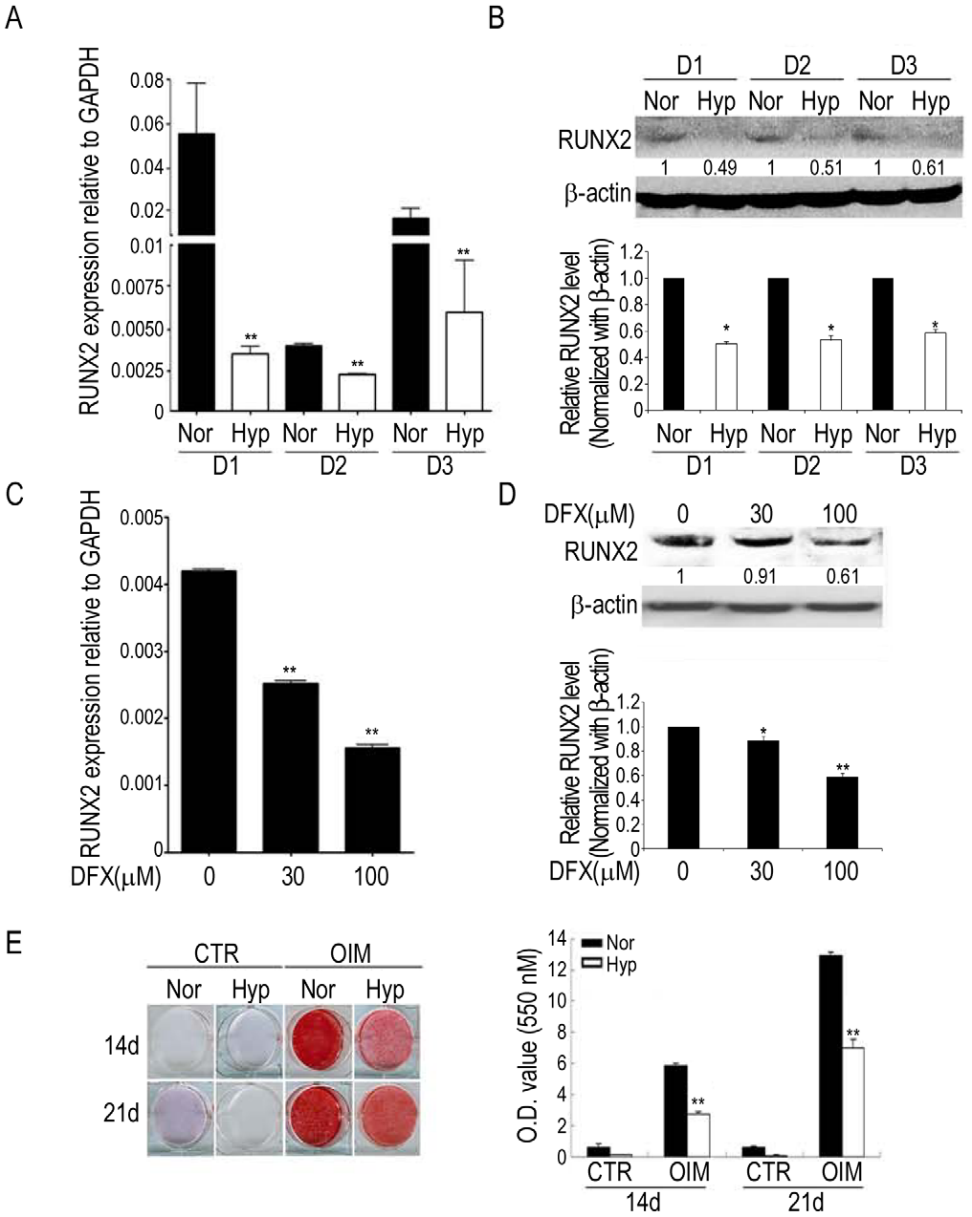
Hypoxia inhibits the expression of RUNX2 and the mineralization capacity of MSCs and zebrafish. MSCs form three individuals (D1, D2, and D3) were induced in OIM under normoxia (Nor) or hypoxia (Hyp) (**A, B**) or treated with indicated concentration of DFX for 3 days (**C, D**) (n = 3). Cells were analyzed by quantitative RT-PCR (**A, C**) and Western blotting (**B, D**). Results are shown as the mean ± SD values. Significance was determined by Student's t-test. (* p<0.05 and ** p<0.01 versus Nor or without DFX). **E**, MSCs without (CTR) or with induction in OIM under normoxia or hypoxia for 14 or 21 days were stained by Alizarin Red S (ARS) and quantification of staining was performed by optic density (O.D.) measurement at O.D.550 nm (n = 3).

To understand the effects of hypoxia on osteogenic differentiation, we induced bone marrow MSCs from three individual donors in osteogenic induction medium (OIM) under normoxia (21% O_2_) and hypoxia (1% O_2_). The expression of *RUNX2* was detected at 3 days of differentiation and the expression level was greater under normoxia than hypoxia both as mRNA (Figure 1A) and protein (Figure 1B) in all three MSCs. The iron chelator desferrioxamine (DFX) has been shown to mimic hypoxic state in regulating several hypoxia-responsive genes [21]. Similarly, decreased *RUNX2* expression was also noted in cells treated with DFX (Figure 1C, D). Further both hypoxia and DFX induced a decrease in the expression of RUNX2 downstream target genes, such as *alkaline phosphatase (AP)*, *bone sialoprotein (BSP)*, *collagen type I alpha 1 (COL1A1)*, *osteopontin (OP)* and *osteocalcin (OC)*, (Figure S1). Induction under hypoxia also had an inhibitory effect on the functional mineralization of MSCs both at 14 and 21 days of osteogenic differentiation (Figure 1E).

### Hypoxia inhibits expression of type1 RUNX2 and its downstream targets

**Figure 2:**
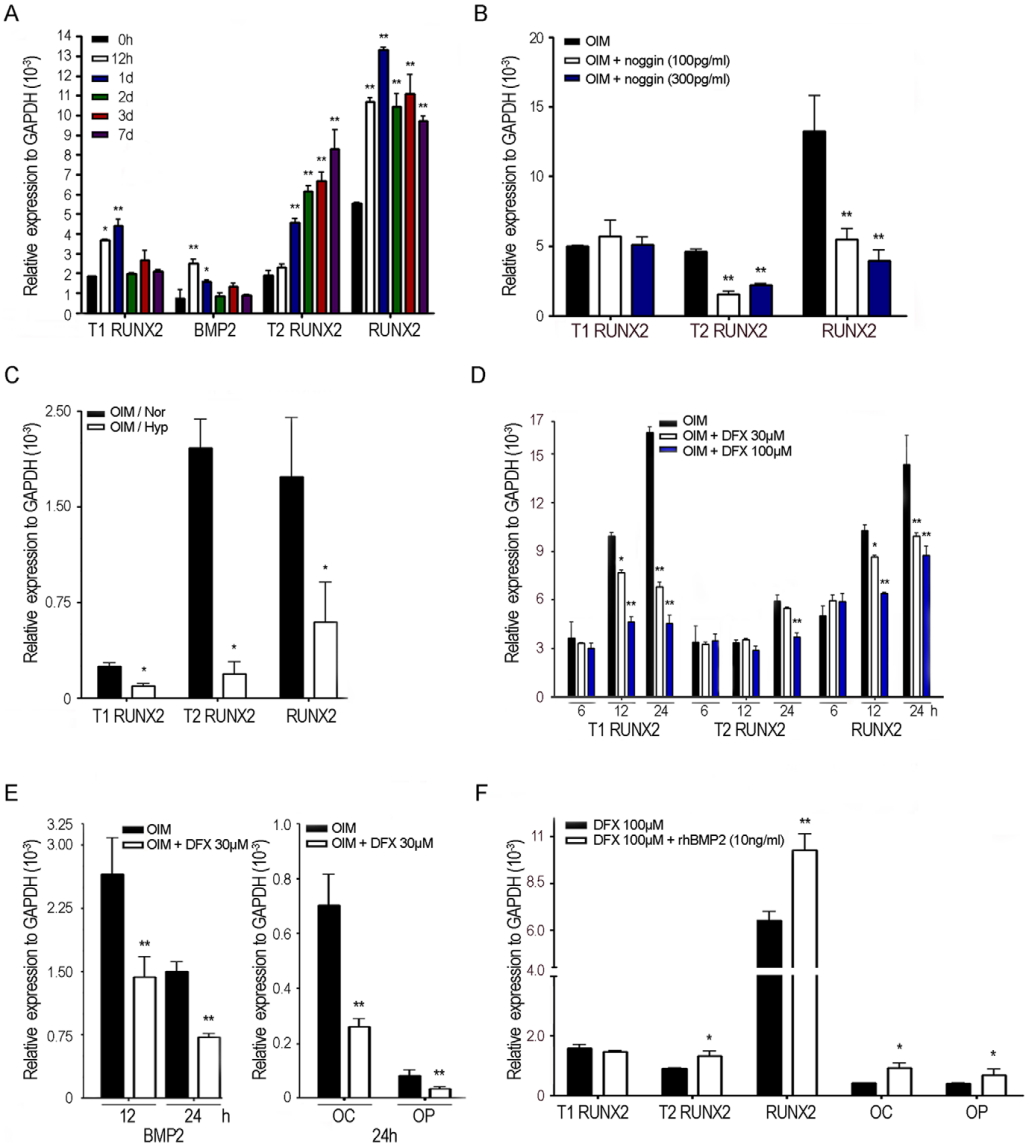
Expression of Type1 and Type2 RUNX2 in MSCs induced for osteogenic differentiation. Quantitative RT-PCR for mRNA levels of indicated genes. **A,** MSCs were induced in OIM for indicated time period (n = 3). **B,** MSCs were induced in OIM without or with noggin treatment for 24 h (n = 3). **C,** MSCs were induced in OIM under normoxia (Nor) or hypoxia (Hyp) for 3day (n = 3). **D, E,** MSCs were induced in OIM or with indicated concentration of DFX for indicated time period (n = 3). **F,** MSCs were induced in OIM in the presence of DFX without or with rhBMP2 for 24 h (n = 3). Results are shown as the relative expression to GAPDH (mean ± SD), and significance was determined by Student's t-test. (* p<0.05 and ** p<0.01 versus black bar).

Similar to induction by FGF2 [9], MSCs induced in OIM that contains dexamethasone, increased in *T1 RUNX2* and *BMP2* expression as early as 12 h after induction, followed by a delayed increase in the expression of *T2 RUNX2* at 24 h (Figure 2A). Interestingly, noggin, a BMP2 antagonist, blocked the increase in *T2* but not *T1 RUNX2* expression (Figure 2B), suggesting that *T1 RUNX2* upregulated *T2 RUNX2* expression occurred through *BMP2*. To explore the key molecule that hypoxia or DFX targeted to regulate osteogenesis, we first found both the expression of *T1 and T2 RUNX2* were downregulated at 3 days under hypoxia (Figure 2C). When examining the early effect of hypoxia, which could be achieved with DFX treatment, we found *T1 RUNX2* and *BMP2* were downregulated as early as 12 h, while *T2 RUNX2*, *OC and OP* were only downregulated at 24 h after treatment (Figure 2D, E). Further, the presence of BMP2 inhibited the DFX-mediated downregulation of *T2 RUNX2*, *OC* and *OP* but not *T1 RUNX2* (Figure 2F).

### Hypoxia downregulates type1 RUNX2 through the HIF-1α-TWIST pathway

**Figure 3:**
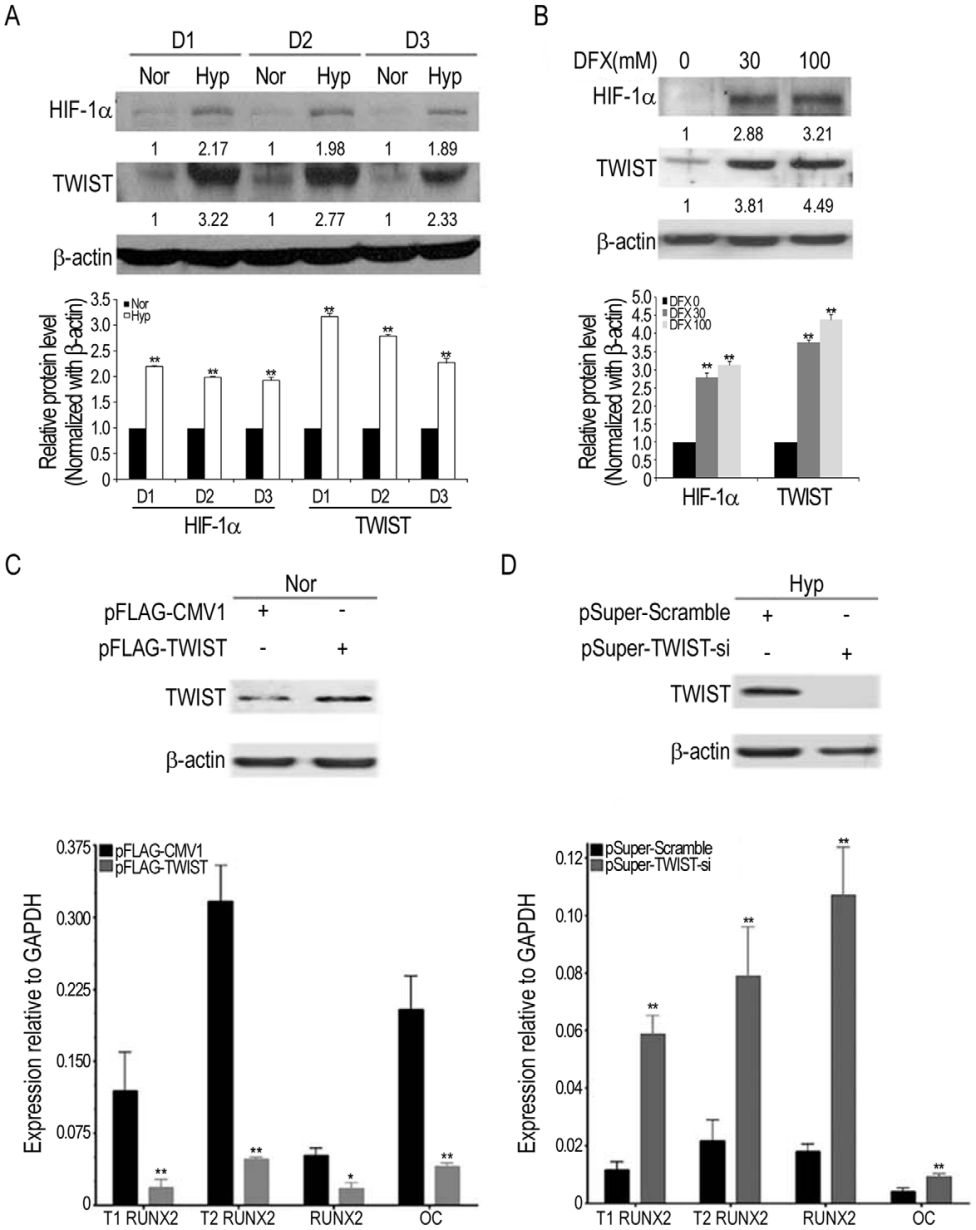
Hypoxia inhibits osteogenesis by MSCs through the HIF-1α-TWIST pathway. **A, B,** MSCs form three individuals (D1, D2, and D3) were induced in OIM under normoxia (Nor) or hypoxia (Hyp) or treated with indicated concentration of DFX for 3 days. Cells were analyzed by Western blotting. **C,** MSCs were transfected with control pFLAG-CMV1 or pFLAG-TWIST vector followed by induction in OIM without DFX treatment (Nor) for 2 days (n = 3). **D,** MSCs were transfected with control pSuper-Scramble or pSuper-TWIST-si vector followed by induction in OIM in the presence of 100 µM DFX (Hyp) for 2 days (n = 3). Cells were assayed by Western blotting and quantitative RT-PCR. Results are shown as the mean ± SD values, and significance was determined by Student's t-test. (* p<0.05 and ** p<0.01 versus control vector).

To examine the molecular mechanism that hypoxia mediated to inhibit osteogenesis, we first demonstrated both HIF-1α and TWIST were upregulated under hypoxia and DFX treatment (hypoxia mimic condition) in MSCs from three individuals (Figure 3A, B). To assess the role of TWIST in regulating differentiation into osteoblasts, MSCs were transfected with overexpression construct, pFLAG-TWIST (control: pFLAG-CMV1), or shRNA construct, pSuper-TWIST-si (control: pSuper-Scramble) in OIM both without and with 100 µM DFX (Hyp) for 2 days. Interestingly, overexpression of TWIST suppressed the expression of *T1*, *T2 RUNX2*, *total RUNX2* and *OC* under normoxia (Figure 3C), while a small effect was noted under hypoxia (Figure S2A). In contrast, knockdown of TWIST stimulated the expression of these genes under hypoxia (Figure 3D), while a small effect was noted under normoxia (Figure S2B). These results suggested the HIF-1α-TWIST pathway regulates osteogenesis by MSCs.

### TWIST suppresses type 1 RUNX2 promoter activity

**Figure 4:**
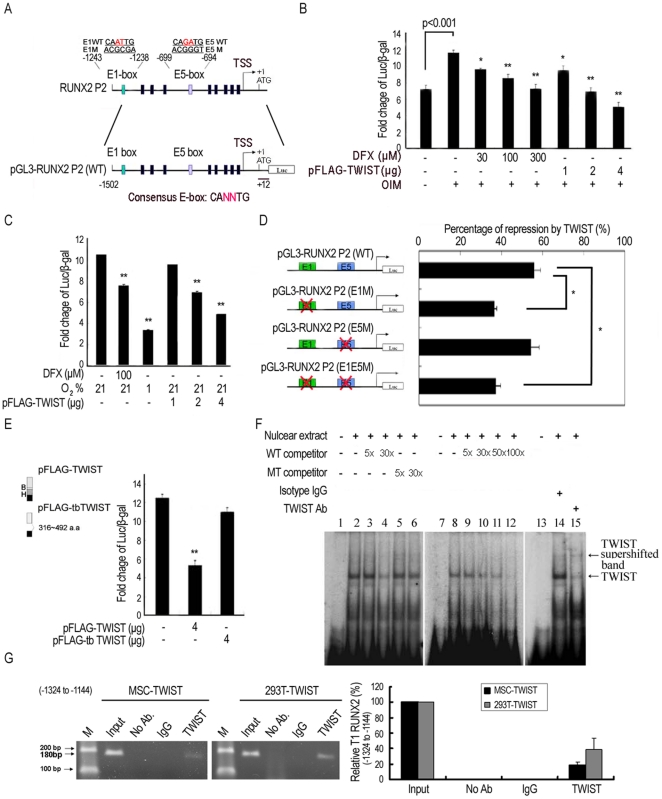
TWIST inhibits Type 1 RUNX2 transcription via binding to its promoter. **A,** Genomic organization of the region flanking the promoter region of human RUNX2 P2 (upper panel) and the schematic representation of the pGL3-RUNX2 P2 reporter construct. Transcription start site, TSS. (**B, C**) Reporter assays showing, in a MSC cell line (**B**) or 293T (**C**), DFX, TWIST and exposure to hypoxia repress the RUNX2 P2 promoter activity in a dose dependent manner (n = 3). β-galactosidase was used as a control of transfection efficiency. **D,** Mutational analysis of E1-box and E5-box sites in the RUNX2 P2 promoter in 293T cells. Reporter constructs containing wild-type RUNX2 P2 (WT), E1-box (E1M) or E5-box (E5M) mutations, or double mutations (E1E5M) were generated and used to analyze the importance of these sites in mediating repression by TWIST (n = 3). **E,** Truncation of the bHLH (basic and helix loop helix) domain (tb TWIST) inhibits TWIST-mediated repression of RUNX2 P2 promoter activity. (n = 3). Each ratio was normalized to the control (pGL3 basic vector), and significance was determined by Student's t-test. (* p<0.05 and ** p<0.01 versus control without DFX, TWIST or exposure to hypoxia). EMSA and ChIP assays demonstrate TWIST binds to the E-box motif in the RUNX2 P2 promoter (−1324 to −1144). **F,** Oligonucleotides for EMSA were the 28 bp probe from the RUNX2 P2 promoter, which contained a TWIST E1-box sequence (TEB). Nuclear extracts prepared from 293T cells, transiently transfected with pFLAG-TWIST, were incubated with [α-32P]-CTP-labelled probe before electrophoresis. No protein extracts: (lane 1, 7 and 13). Competition assays were performed in the presence of excess wild type (WT) (lane 3, 4, 9–12) or mutation type (MT) (lane 5 and 6) of unlabelled oligonucleotides containing the TBE. The supershift assay was performed in the presence of an isotype (lane 14) or anti-TWIST antibody (lane 15), and the position of supershifted bands is indicated (arrow). **G,** ChIP analysis of MSCs and 293T after transfection of pFLAG-TWIST. The two chromatins were incubated either without antibodies, with an anti-TWIST antibody or with isotype IgG antibody. The two samples with 180 bp fragment of the E1-box in the RUNX2 P2 promoter were amplified by PCR (left panel) and were also quantified with quantitative RT-PCR (right panel). Input, 2% of total input lysate. Results are shown as the mean ± SD values.

To demonstrate whether RUNX2 was directly regulated by TWIST during osteogenesis, analysis of the human *RUNX2 P2* (for *T1 RUNX2*) and *P1* (for *T2 RUNX2*) promoter activity was performed using luciferase reporter constructs in a MSC cell line. We first revealed the upregulation of *P2* (Figure 4A, B) but not *P1* promoter activity (Figure S3A) upon induction in OIM, while the *P1* promoter activity, consistent with a previous report [22], was stimulated by overexpression of β-catenin [23] (Figure S3A) Interestingly, DFX treatment or overexpression of TWIST abrogated the upregulation of *P2* promoter in a dose dependent manner (Figure 4B). Similarly, DFX treatment or hypoxia or overexpression of TWIST in 293T cells also downregulated the promoter activity of *P2* in a dose-dependent manner (Figure 4C). However, DFX treatment or overexpression of TWIST did not inhibit β-catenin-induced increase in the *P1* promoter activity (Figure S3A). These data suggest TWIST directly inhibited *P2* promoter activity in MSCs undergoing osteogenic differentiation.

### TWIST directly binds to RUNX2 P2 promoter

TWIST is reported to bind to the E-box motifs to regulate transcription [24]. To identify the minimum promoter region required for inhibition by TWIST, a series of *5′ P2 promoter* deletion constructs were generated. Deletions of the −1502 to −1232, but not other regions, significantly inhibited the suppressive effect of TWIST on the promoter activity (Figure S3B). These findings suggest the existence of a TWIST-responsive sequence in the −1502 to −1232 promoter region. To further find where TWIST binds to pGL3-RUNX2 P2 (WT) promoter, site-directed mutagenesis of the putative E-box in the *P2* promoter prevented suppression under TWIST was examined. The pGL3-RUNX2 P2 (E1M) (distal first E-box mutation) and pGL3-RUNX2 P2 (E1E5M) (first and fifth E-boxes double mutations) of the *P2* promoters significantly inhibited the TWIST-mediated suppression of the promoter activity in a same degree in 293T, while pGL3-RUNX2 P2 (E5M) did not inhibit any change on TWIST effect (Figure 4D), suggesting the E1-box in the *P2* promoter was the binding site for TWIST. To determine the E1-box binding domain in TWIST, a construct with truncated 316–492 a.a basic helix-loop-helix (bHLH), pFLAG-tbTWIST, was transfected to 293T, truncation of the bHLH domain abrogated the inhibition of *P2* promoter activity by TWIST (Figure 4E).

Electrophoretic mobility shift assays (EMSAs) using an oligonucleotide containing the E1-box sequence from *RUNX2 P2* promoter incubated with nuclear extracts of TWIST-overexpressing 293T cells demonstrated the direct binding of TWIST to the wild-type probe (TWIST E1-box sequences (TEB)) (Figure 4F, lane 2, 8 and 14 v.s. no nuclear extract controls at Figure 4A, lane 1, 7 and 13). Competition by unlabelled wild type probe abolished the binding activity (Figure 4F, lane 3–4, 9–12), but the mutated probe did not show the same effect (Figure 4F, lane 5 and 6). Addition of anti-TWIST antibodies, rather than isotype antibodies, into TWIST-overexpressing nuclear extracts showed a supershifted band in gel electrophoresis (Figure 4F, lane 15). To investigate whether TWIST is directly associated with the *RUNX2 P2* promoter via binding to E1-box, we performed chromatin immunoprecipitation (ChIP). PCR amplification (Figure 4G, left panel) and real-time PCR (Figure 4G, right panel) showed that the fragments containing the E1-box (180 bp) were immunoprecipitated with TWIST antibody in a MSC cell line and 293T after transfection of pFLAG-TWIST. However, an internal control of RUNX2 promoter (−943 to −878) that does not contain the binding site (Figure S4A) and the unrelated promoters of GAPDH (Figure S4B), BCL-2 (Figure S4C) and NKG2A (Figure S4D) were not immunoprecipitated with TWIST antibody. These data taken together suggest TWIST downregulated *P2* promoter activity by direct binding through the bHLH domain to the E1-box in the *P2* promoter.

## Discussion

Oxygen (O_2_) is a substrate for energy production in the cell and is a rapid regulator of cellular metabolism. Recent studies have also implicated O_2_ and its signal transduction pathways in controlling cell proliferation, fate, and morphogenesis during the development of many tissues [25], including the skeletal system [26], [27]. Hypoxia has recently been reported by us and others to inhibit osteogenesis in MSCs and osteoblast [18], [28], [29], however the detailed mechanism underlying the inhibition effects of hypoxia on osteogenesis is unknown. In the current study, we discovered TWIST, a downstream target of HIF-1α, acts as a transcription repressor of RUNX2 through binding to the E-box located on the promoter of *T1 RUNX2*, leading to the suppression of *T1 RUNX2*, followed by suppression of *BMP2*, *T 2 RUNX2* and downstream targets of *RUNX2* in MSCs.

Previously, *T2 RUNX2* was considered important for osteogenesis. Its expression is induced upon stimulation with BMP2 or activation of the canonical WNT and β-catenin/TCF1 pathways [22]. However, the β-catenin-induced increase in *P1* promoter activity was not suppressed by DFX treatment or overexpression of TWIST, suggesting hypoxia or TWIST did not inhibit *P1* promoter activity in MSCs undergoing osteogenic differentiation. Interestingly, there are four E-boxes in the *P1* promoter. Therefore it would be interesting to clarify why TWIST did not inhibit the *P1* promoter activity. Although *T1 RUNX2* is constantly expressed in various cells including osteoblast, its expression is seldom, if ever, emphasized in osteogenesis and bone formation [30]. The current data first demonstrated the induction of *T1 RUNX2* in MSCs undergoing osteogenesis, and found a putative TWIST binding site, E1-box, in the *RUNX2 P2* promoter. To our knowledge, it has not been demonstrated before that *T1 RUNX2* is an important target for controlling osteogenesis by hypoxia or HIF-1α-TWIST, an important environment or signaling occurred during a lot of pathophysiological conditions associated with normal development and regeneration, or acquired and genetic diseases. Therefore, signaling pathways or molecules that control the transcription of *T1 RUNX2* may be modified to control MSC osteogenesis upon application of these cells in treating bone diseases especially associated with ischemia and hypoxic conditions.

The roles of HIF proteins and downstream signaling pathways in skeletal development have recently been studied. The HIF-1 pathway has been identified as a key component in the coupling of angiogenesis and osteogenesis during bone development and regeneration [26], [27]. Mice overexpressing Hif-1α in osteoblasts through selective deletion of the von Hippel-Lindau gene (Vhl) expressed high levels of Vegf and developed extremely dense, heavily vascularized long bones. By contrast, mice lacking Hif-1α in osteoblasts had the reverse skeletal phenotype of that of the Vhl mutants both during skeletal development [27] and bone repairing process [26]. Moreover, HIF-2α, independently of oxygen-dependent hydroxylation, has recently been reported essential for endochondral ossification of cultured chondrocytes and embryonic skeletal growth, and important for development of osteoarthritis in mice [31]. However, manipulation of HIF-1α levels in osteoblasts in the calvarial bones did not influence the formation of the flat bones of the skull [32]. In the current study, we found HIF-1α activated TWIST, which in turn repressed transcription of RUNX2 and inhibited osteogenic differentiation in MSCs. Consistent with the *in vitro* study, exposure of zebrafish to hypoxia or hypoxia-mimicking agent also induced the hif-twist pathway and inhibited the *in vivo* bone formation [2nd paper]. Thus, the effects of HIF pathways on *in vitro* osteogenesis and *in vivo* bone formation are context and situation-specific.

Bialek et al. demonstrated Twist inhibited DNA binding and gene activation by Runx2 via the interaction with Runt domain through a Twist box in the carboxy-terminus, while Runx2 expression was not affected in mice carrying Twist heterozygosity [24]. The current data found overexpression and knockdown of TWIST increased and decreased the expression of RUNX2 both as mRNA and protein, respectively. The discrepancy between these two studies may be due to the suppression of Twist expression by Twist heterozygosity in mice is not sufficient to downregulate Runx2. The negative role of TIWST in regulating osteogenesis has also been implicated in phenotypic abnormalities seen in human disorders with TWIST mutation, such as Saethre–Chotzen syndrome [33], [34], an autosomal dominant disorder with characteristic abnormalities in craniosynostoses resulting from premature closure of cranial sutures, short stature, and developmental limb deformities (syndactyly and polydactyly). Future exploration of the TWIST signaling pathways may help in developing strategies to control osteogenesis and skeleton development through RUNX2 suppressions. In conclusion, these data suggest the important roles of TWIST in regulating RUNX2 expression and controlling MSC osteogenesis. Further exploration of the molecules or signaling pathways that are involved in TWIST regulation may provide new strategies for modifying these cells in treating fracture healing and bone reconstruction.

## Methods

### Cell culture and oxygen deprivation

The Primary MSCs were gifted by Dr. D.P. Prockop, isolated as previously described [35] and grown in CCM [MEM (Gibco) with 16.6% FBS, 100 U/ml penicillin, and 10 µg/ml streptomycin] at 37°C under 5% CO_2_ atmosphere. The medium was changed twice a week and subcultured at 1∶5 at subconfluence. For hypoxic conditions, cells were cultured in a gas mixture composed of 94% N_2_, 5% CO_2_, and 1% O_2_ or treated in medium containing 30 or 100 mM of DFX that mimics the hypoxic conditions by inhibiting the hydroxylation of a prolyl residue that is essential for the ubiquitination of HIF-1α.

### Induction and histochemical studies

For osteogenic differentiation, cells were reseeded at 10^4^ cells/cm^2^ and induced in osteogenic induction medium [OIM: α MEM supplemented with 16.6% FBS, 50 µg/ml ascorbate-2 phosphate (Nacalai, Kyoto, Japan), 10^−8^ M dexamethasone (Sigma) and 10 mM β-glycerophosphate (Sigma)] under normoxic and hypoxic conditions or treated with DFX. After induction in OIM, cells were fixed and stained with Alizarin Red S (Sigma) for 30 min to show osteogenic differentiation. Following wash five times with PBS, stained cultures were photographed and quantification of calcium deposition was made by extracting Alizarin Red S staining with 10% cetylpyridinium chloride and measuring the O.D. of the extract at 550 nm using an ELISA reader (Sepctra MAX 250; Molecular Devices, Sunnyvale, CA).

### Plasmid constructions

The pFLAG-CMV1, pFLAG-TWIST, pSuper-scramble-si and pSuper-TWIST-si plasmids were gifts from Muh-Hwa Yang (University of National Yang-Ming University) [16]. The pSG5HA β-catenin and pRC/CMV-LacZ plasmids were gifts from Fung-Fang Wang (University of National Yang-Ming University) [12], [23]. The human RUNX2 P1 promoter (−767∼+31) and P2 promoter (−1502∼+12) were constructed by PCR amplification of the 293T DNA using the primer pairs:

pGL3-RUNX2 P1 F′: 5′-GTGGTACCGAATAATTTCAGCATT-3′ and pGL3-RUNX2 P1 R′: 5′-TGAGCTCCCAGTACAAGAGTTTT-3′; pGL3-RUNX2 P2 F′: 5′-GTGGTACCAGGAAGGCTTTATTGATT-3′ and pGL3-RUNX2 P2 R′: 5′-GGAGCTCCCTACATAAAACAGGAAAC-3′. Each fragment of the pGL3-RUNX2 P1 (799 bp) or pGL3-RUNX2 P2 (1515 bp) promoter was subcloned into the pGL3 vector (Promega). A series of 5′ promoter deletion constructs (−1232∼+12, −1032∼+12, −852∼+12 and −687∼+12) were generated by PCR and cloned into the *KpnI-SacI* sites of the pGL3 basic vector. The primer pairs used included −1232 F′:5′-GC GGTACC TATTGGGAACATGGAATT-3′; −1032 F′:5′-GC GGTACC AGGAAGACATGGAAATAA-3′; −852 F′:5′-TT GGTACC TTAGCTGACTCAGGTTAAA-3; −687 F′:5′-GC GGTACC GACACAAGACATAATAGAAC-3; +12 R′:5′-G GAGCTC CCTACATAAAACAGGAAAC-3′. Underlined nucleotides indicate restricftion enzyme sites. The pGL3-RUNX2 P2 E1-box and E5-box mutants were generated from pGL3-RUNX2 P2 (WT) using Gene Tailor™ Site-Directed Mutagenesis System (Invitrogen) with the following primer pairs: E1WT:5′-AGAAATCTACTGTAATATGCCAATTGTATTGGGAAC-3′ and 5′-GCATATTACA GTAGATTTCT TAACAGTGTG-3′-; E1M: 5′-AGAAATCTACTGTAATATGCACGCGATATTGGGAAC-3′ and 5′-GCATATTACA GTAGATTTCT TAACAGTGTG-3′ E5WT:5′-AGTATGTCATTCCAGGATGGCAGATGGGACACAAGA-3′ and 5′-CCATCCTGGA ATGACATACT TGACTGCTTA-3′; E5M:5′-AGTATGTCATTCCAGGATGGACGGGTGGACACAAGA-3′ and 5′-CCATCCTGGA ATGACATACT TGACTGCTTA-3. Double underlined nucleotides indicate wild-type and mutated E-boxes. The PCR products, were subcloned into the KpnI-SacI sites of the pGL3 basic vector to create pGL3-RUNX2 P2 (E1M), (E5M) and (E1E5M) constructs. The pFLAG-tbTWIST plasmid was generated by inserting a 537 bp fragment, which truncated the bHLH (342∼518th) of pFLAG-TWIST (equal to 316∼492th of human TWIST mRNA), into the *EcoRI/BamHI* sites of the pFLAG-CMV1 vector.

### RNA extraction and quantitative RT-PCR

Total RNA was prepared by using the Trizol reagent (Invitrogen) according to the manufacturer's specifications. First strand cDNA was synthesized using Superscript III (Invitrogen), Random primer (Invitrogen), 10 mM DTT (Invitrogen), and RNaseOUT ribonuclease RNase inhibitor (Invitrogen). The protocol of quantitative RT-PCR was performed using cDNA as the template in a 20-µl reaction mixture containing FastStart SYBR Green Master (Roche Applied Science) and a specific primer pair of each cDNA according to the published sequences:

Human GAPDH F′: CTCTGCTCCTCCTGTTCGACA

Human GAPDH R′: ACGACCAAATCCGTTGACTC

Human ALK-P F′: CCCAAAGGCTTCTTCTTG

Human ALK-P R′: CTGGTAGTTGTTGTG AGCAT

Human BMP2 F′: TTCCACCATGAAGAATCTTTGGA

Human BMP2 R′: CCTGAAGCTCTGCTGAGGTGAT

Human BSP F′: AACCTACAACCCCACCACAA

Human BSP R′: AGGTTCCCCGTTCTCACTTT

Human COL1A1 F′: GACATGCTCAGCTTTGTGGA

Human COL1A1 R′: CTTTGTCCACGTGGTCCTCT

Human OC F′: GACTGTGACGAGTTGGCTGA

Human OC R′: CTGGAGAGGAGCAGAACTGG

Human OP F′: TTTTCTGGATCCTCCATTGC

Human OP R′: CAAAAGCCATATGCTGCTCA

Human T1 RUNX2 F′: GGCAGTCGGCCTCATCAAA

Human T1 RUNX2 R′: ACAAGTTAGCGAAGTGGCCG

Human T2 RUNX2 F′: GGTTAATCTCCGCAGGTCAC

Human T2 RUNX2 R′: GTCACTGTGCTGAAGAGGCT

Human RUNX2 F′: GGAGTGGACGAGGCAAGAGTTT

Human RUNX2 R′: AGCTTCTGTCTGTGCCTTCTGG

Analysis of the results were carried out using the software supplied with the ABI Step One Real-Time PCR System machine and each expression was calculated relative to the human GAPDH (delta CT) and then relative to controls (delta delta CT) using the fluorescence threshold of the amplification reaction and the comparative CT method.

### Protein extraction, western blot analysis

The cells were rinsed with PBS and lysed in 0.2 ml of protein extraction reagent (M-PER, Pierce, Rockford, Illinois) plus protease inhibitor cocktail (HaltTM, Pierce) for 5 min on ice. Protein concentrations were determined using the BCA assay (Pierce). After being heated for 5 min at 95°C in a sample buffer, aliquots of the cell lysates were run on a 10% SDS polyacrylamide gel. Proteins were transferred to PVDF membrane filters. The filter was blocked for 1 h with TBS containing 5% nonfat dry milk and 0.05% Tween 20 and then incubated overnight at 4°C with the primary antibodies. The filter was washed 3 times for 10 min each with TBS containing 0.05% Tween 20. Bound primary antibodies were detected by incubating for 1 h with horseradish peroxidaseconjugated goat anti-mouse or anti-rabbit IgG (BD PharMingen, San Diego, CA). The filter was washed and developed using a chemiluminescence assay (Perkin Elmer Life Science, Inc., Boston, MA). Densitometric analysis was carried out using Image Master 2D Elite software (Amersham Biosciences). Anti human HIF-1α (H-206, sc-10790), TWIST (H-81, sc-15393), RUNX2 (C-19, sc-8566) and ACTIN (I-19, sc-1616) antibodies were purchased from Santa Cruz Biotechnology.

### Transfections

Transfections were performed by electroporation using the Nucleofector system (Amaxa Biosystems, Koln, Germany). In brief, 4×10^5^ cells were resuspended in 80 µl Human MSC Nucleofector solution (Amaxa Biosystems) at room temperature followed by addition of 20 µl of Human MSC Nucleofector solution containing 4 µg of DNA The transfection was carried out under the program C-17 of the Nucleofector device, as recommended by the manufacturer. The transfected cells were then suspended in an appropriate volume of 20% FBS supplemented DMEM-LG medium and seeded for further culture. After 24 h of incubation, the medium was replaced by OIM and cells were cultured for another 2 days without or with DFX. The efficiency of transfection as evaluated by the expression of EGFP in cells transfected with pMaxGFP vector was more than 50%.

### Transient transfection and luciferase assays

The reporter constructs were co-transfected into human embryonic kidney cancer cells, 293T (ATCC-CRL-11268, Manassas, VA) or immortalized MSC line with different expression vectors and internal control plasmids using Lipofetamine 2000 reagent (Invitrogen) under normoxic, hypoxic or hypoxia-mimicking conditions. At 48 h post transfection, enzyme activity was measured using the Dual-Luciferase Reporter Assay (Promega).

### Electrophoretic mobility shift assay (EMSA)

Nuclear extracts were obtained from 293T cells transfected with pFLAG-TWIST. The specific activity of the [α-^32^p]-labeled probes used in the assays was adjusted to 10^4^ CPM/1.25 pmole DNA. The probe was incubated at 25°C for 30 min with 5 µg of nuclear extract in a buffer consisting of 10 mM Tris-HCl, pH 7.5, 50 mM KCl, 5 mM dithiothreitol, 10% glycerol, 1 µg poly dI/dC), bromophenol blue/50% glycerol in a total volume of 20 µl. Samples were loaded on 5% polyacrylamide gels and run at 150 V for 2 h in 1× TBE buffer. The gels were dried and exposed to x-ray film. All unlabeled competitor oligonucleotides were added before incubation with labeled oligonucleotides. For supershift assay, anti-human TWIST polyclonal antibody (1 µg) (Abcam, ab50581) was added at 4°C for 1 day before the addition of labeled oligonucleotides. Densitometric analysis of the DNA-protein complexes was performed on the captured images using the typhoon 8600 and ImageMaster VDS software (Amersham Biosciences).

### Chromatin immunoprecipitation assay (ChIP)

To demonstrate the binding of TWIST protein to the E1-box of *RUNX2 P2* promoter, the ChIP assay was performed with a commercial kit (Upstate Biotechnology; Lake Placid, NY; www.upstatebiotech.com) using the manufacturer's protocol with minor adjustments. The MSCs and 293T transfected with pFLAG-TWIST were grown to confluence, and formaldehyde was added directly to the culture medium at a final concentration of 1% followed by incubation for 20 min at 37°C.The cells were washed at 4°C in PBS, lysed on ice for 10 min in lysis buffer [10 mM Tris HCl, pH 8.0, 1% SDS] containing phosphatase and protease inhibitors. The lysates were sonicated three times for 30 sec under optimized conditions to produce average DNA fragments of 1 kb (Branson Sonifier 450), and the debris was removed by centrifugation. The supernatant was split into several aliquots. One aliquot of the soluble chromatin was saved at −20°C for preparation of input DNA, and the remainder was diluted 10 times in immunoprecipitation (IP) buffer [10 mM Tris HCl, pH 8.0, 0.1% SDS, 1% Triton X-100, 1 mM EDTA, and 150 mM NaCl] containing phosphatase and protease inhibitors, and incubated overnight (4°C) with polyclonal antibody to human TWIST (H-81, sc-15393) (Santa Cruz Biotechnology). DNA–protein complexes were isolated on salmon sperm DNA linked to protein A agarose beads and eluted with 1% SDS, and 0.1 M NaHCO3. Cross-linking was reversed by incubation at 65°C for 5 h. Proteins were removed with proteinase K, and DNA was extracted with phenol/chloroform, redissolved and PCR-amplified with E1-box of T1 RUNX2 promoter primers F′: 5′- CATAGTGAAGGCGTGTTGC -3′ and R′:5′- GCTACTCCCTCCTTTGTCAAG-3′; which gave a product length of 180 bp. The primers for the internal control of T1 RUNX2 (−943 to −878) promoter are F′: 5′- GGGCAGGATCTTGGCAATG -3′ and R′: 5′-GCTGGTGCAGATCCTTCACTG-3′ (product: 66 bp). The primers for the unrelated promoters of GAPDH are F′: 5′-TTGAACCAGGCGGCTGCGGA-3′ and R′:5′-GGAGGCTGCG GGCTCAATTT-3′ (product: 188 bp); the primers for the unrelated promoters of BCL-2 are F′: 5′-TTGTAGTGTGTATGCCCTG-3and R, 5′-CGGAACACTTGATTCTGGTG-3′(product: 162 bp); and the primers for the unrelated promoters of NKG2A are F′: 5′- ACCAACTAAGTGACACACTTTC-3′and R: 5′- AGGAAATTCTACACATGGGC-3′ (product: 151 bp). The cycling parameters were 35 cycles, with each cycle consisting of denaturing at 94°C for 30 sec, annealing at 60°C for 30 sec, and elongating at 72°C for 30 sec, with additional 10 min incubation at 72°C after completion of the last cycle. The TWIST binding site was detected in E1-box of the T1RUNX2. All resulting precipitated DNA samples were also quantified with quantitative RT-PCR. Data were expressed as the percentage of input DNA.

## Supporting Information

Figure S1 **Hypoxia inhibits the expression of RUNX2 downstream genes in MSCs induced for osteogenic differentiation.** MSCs were induced in OIM under normoxia (Nor) or hypoxia (Hyp) (**A–E**) or treated with DFX (**F–H**) at indicated concentration for 3 days. Cells were analyzed by quantitative RT-PCR for downstream genes of RUNX2, such as *alkaline phosphatase (ALK-P)*, *bone sialoprotein (BSP)*, *collagen type I alpha 1 (COL1A1)*, *osteopontin (OP)*, and *osteocalcin (OC)* (n = 3). Results are shown as the relative expression to GAPDH (mean ± SD), and significance was determined by Student's t-test. (* p<0.05 and ** p<0.01 versus Nor or without DFX).
(TIF)

Figure S2 **TWIST inhibits RUNX2 expression in MSCs undergoing osteoblast differentiation.** **A**, MSCs were transfected with control pFLAG-CMV1 or pFLAG-TWIST vector followed by induction in OIM in the presence of 100 µM DFX (Hyp) for 2 days (n = 3). **B**, MSCs were transfected with control pSuper-Scramble or pSuper-TWIST-si vector followed by induction in OIM without DFX treatment (Nor) for 2 days (n = 3). Cells were assayed by Western blotting and quantitative RT-PCR. Results are shown as the mean ± SD values, and significance was determined by Student's t-test. (* p<0.05 and ** p<0.01 versus control vector).
(TIF)

Figure S3 **Treatment with DFX and overexpression with TWIST induce no changes in** ***Type 2 RUNX2*** **transcription.** **A**. Genomic organization of the region flanking the promoter region of human RUNX2 P1 (upper panel) and the schematic representation of the pGL3-RUNX2 P1 reporter construct. Transcription start site, TSS. Reporter assays showing, in a MSC cell line, β-catenin but not osteogenic differentiation enhances the RUNX2 P1 promoter activity. Treatment with DFX and overexpression with TWIST do not repress the RUNX2 P1 promoter activity (n = 3). β-galactosidase was used as a control of transfection efficiency. **B**. Deletion analysis of various regions in the RUNX2 P2 promoter. Reporter constructs containing wild-type RUNX2 P2 (−1502), with deletion of −1502∼−1232 (−1232), −1232∼−1032 (−1032). −1032∼−852 (−852) or −852∼−687 (−687) were generated and used to analyze the importance of these regions in mediating the repression of RUNX2 P2 promoter activity by TWIST in 293T cells (n = 3).
(TIF)

Figure S4 **Unrelated fragments were used as internal control in ChIP analysis.** ChIP analysis of MSCs after transfection with pFLAG-TWIST. The chromatin was incubated with anti-TWIST antibody. The internal control of T1RUNX2 promoter (−943 to −878) that does not contain the binding site (Figure S4A) and three unrelated promoters, GAPDH (Figure S4B), BCL-2 (Figure S4C) and NKG2A (Figure S4D) were amplified by PCR (upper panel) and quantified with quantitative RT-PCR (lower panel). Input, 2% of total input lysate. Results are shown as the mean ± SD values.
(TIF)
