# Supplementary figures and images for: Hypoxia Inhibits Osteogenesis in Human Mesenchymal Stem Cells through Direct Regulation of RUNX2 by TWIST
Source: PLoS One. 2011 Sep 9;6(9):e23965. doi: 10.1371/journal.pone.0023965 (PMC3170288; doi:10.1371/journal.pone.0023965)

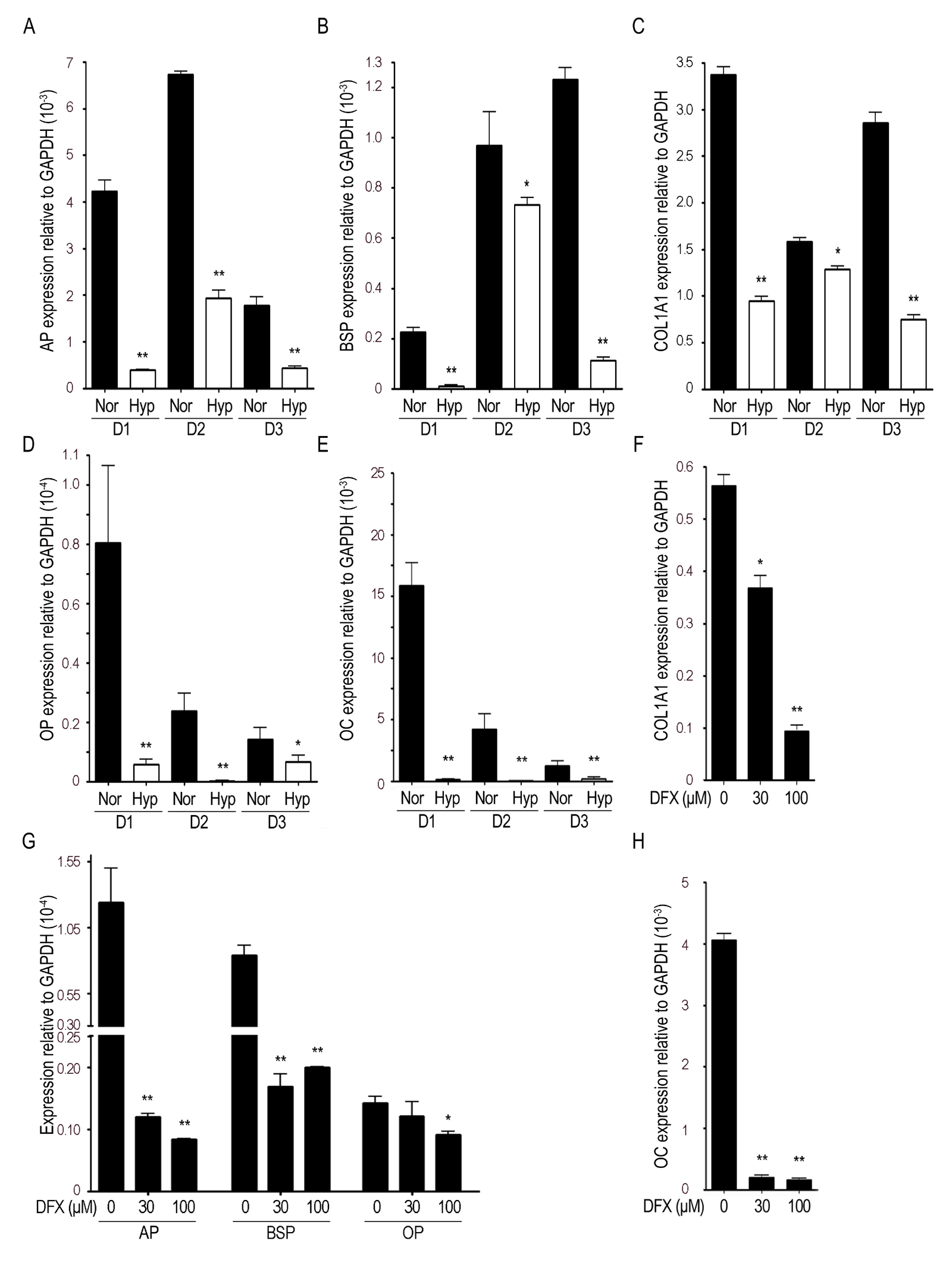

Supplement: Figure S1 — Hypoxia inhibits the expression of RUNX2 downstream genes in MSCs induced for osteogenic differentiation. MSCs were induced in OIM under normoxia (Nor) or hypoxia (Hyp) (A–E) or treated with DFX (F–H) at indicated concentration for 3 days. Cells were analyzed by quantitative RT-PCR for downstream genes of RUNX2, such as alkaline phosphatase (ALK-P), bone sialoprotein (BSP), collagen type I alpha 1 (COL1A1), osteopontin (OP), and osteocalcin (OC) (n = 3). Results are shown as the relative expression to GAPDH (mean ± SD), and significance was determined by Student's t-test. (* p<0.05 and ** p<0.01 versus Nor or without DFX). (TIF) [file pone.0023965.s001.tif]

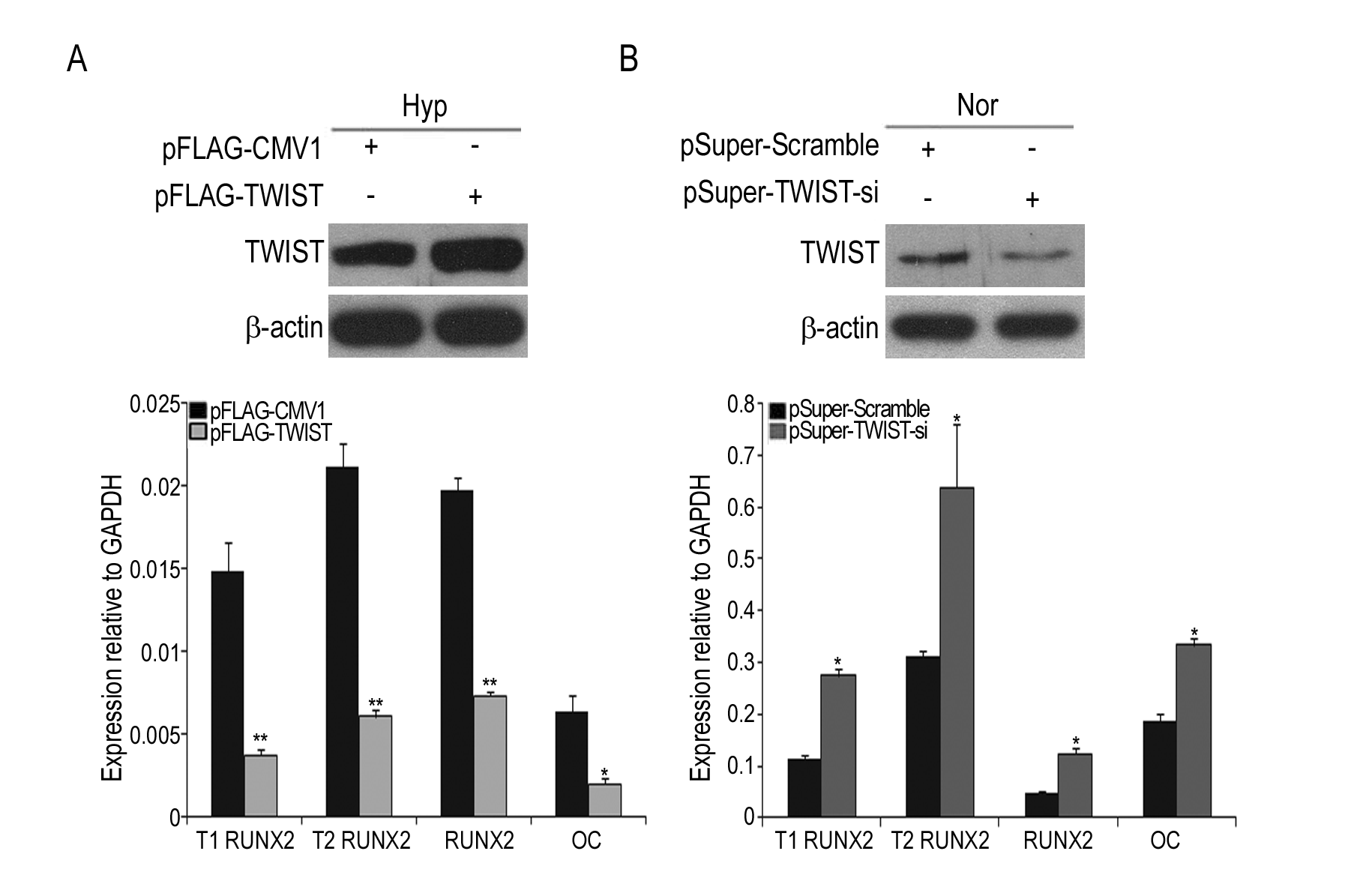

Supplement: Figure S2 — TWIST inhibits RUNX2 expression in MSCs undergoing osteoblast differentiation. A, MSCs were transfected with control pFLAG-CMV1 or pFLAG-TWIST vector followed by induction in OIM in the presence of 100 µM DFX (Hyp) for 2 days (n = 3). B, MSCs were transfected with control pSuper-Scramble or pSuper-TWIST-si vector followed by induction in OIM without DFX treatment (Nor) for 2 days (n = 3). Cells were assayed by Western blotting and quantitative RT-PCR. Results are shown as the mean ± SD values, and significance was determined by Student's t-test. (* p<0.05 and ** p<0.01 versus control vector). (TIF) [file pone.0023965.s002.tif]

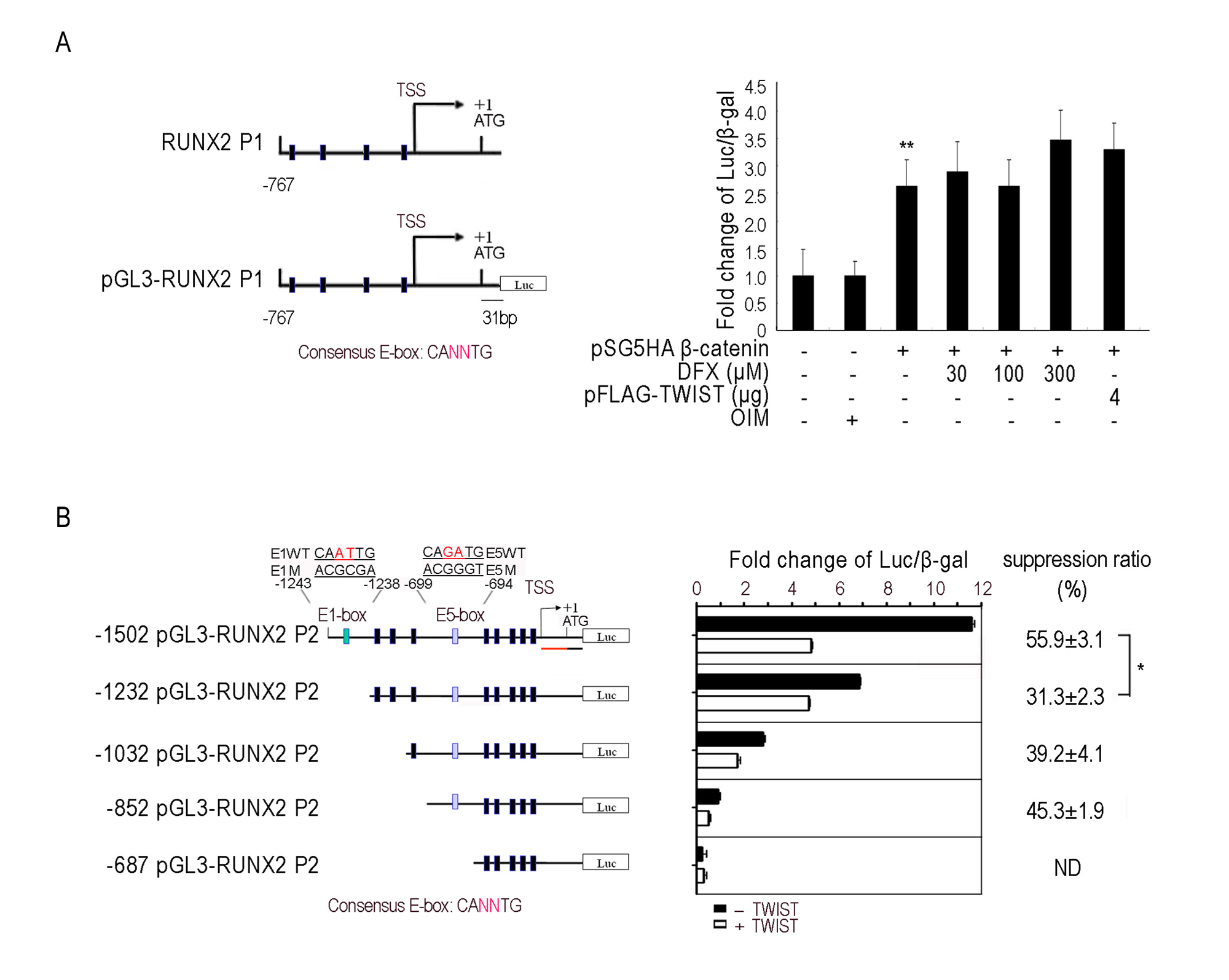

Supplement: Figure S3 — Treatment with DFX and overexpression with TWIST induce no changes in Type 2 RUNX2 transcription. A. Genomic organization of the region flanking the promoter region of human RUNX2 P1 (upper panel) and the schematic representation of the pGL3-RUNX2 P1 reporter construct. Transcription start site, TSS. Reporter assays showing, in a MSC cell line, β-catenin but not osteogenic differentiation enhances the RUNX2 P1 promoter activity. Treatment with DFX and overexpression with TWIST do not repress the RUNX2 P1 promoter activity (n = 3). β-galactosidase was used as a control of transfection efficiency. B. Deletion analysis of various regions in the RUNX2 P2 promoter. Reporter constructs containing wild-type RUNX2 P2 (−1502), with deletion of −1502∼−1232 (−1232), −1232∼−1032 (−1032). −1032∼−852 (−852) or −852∼−687 (−687) were generated and used to analyze the importance of these regions in mediating the repression of RUNX2 P2 promoter activity by TWIST in 293T cells (n = 3). (TIF) [file pone.0023965.s003.tif]

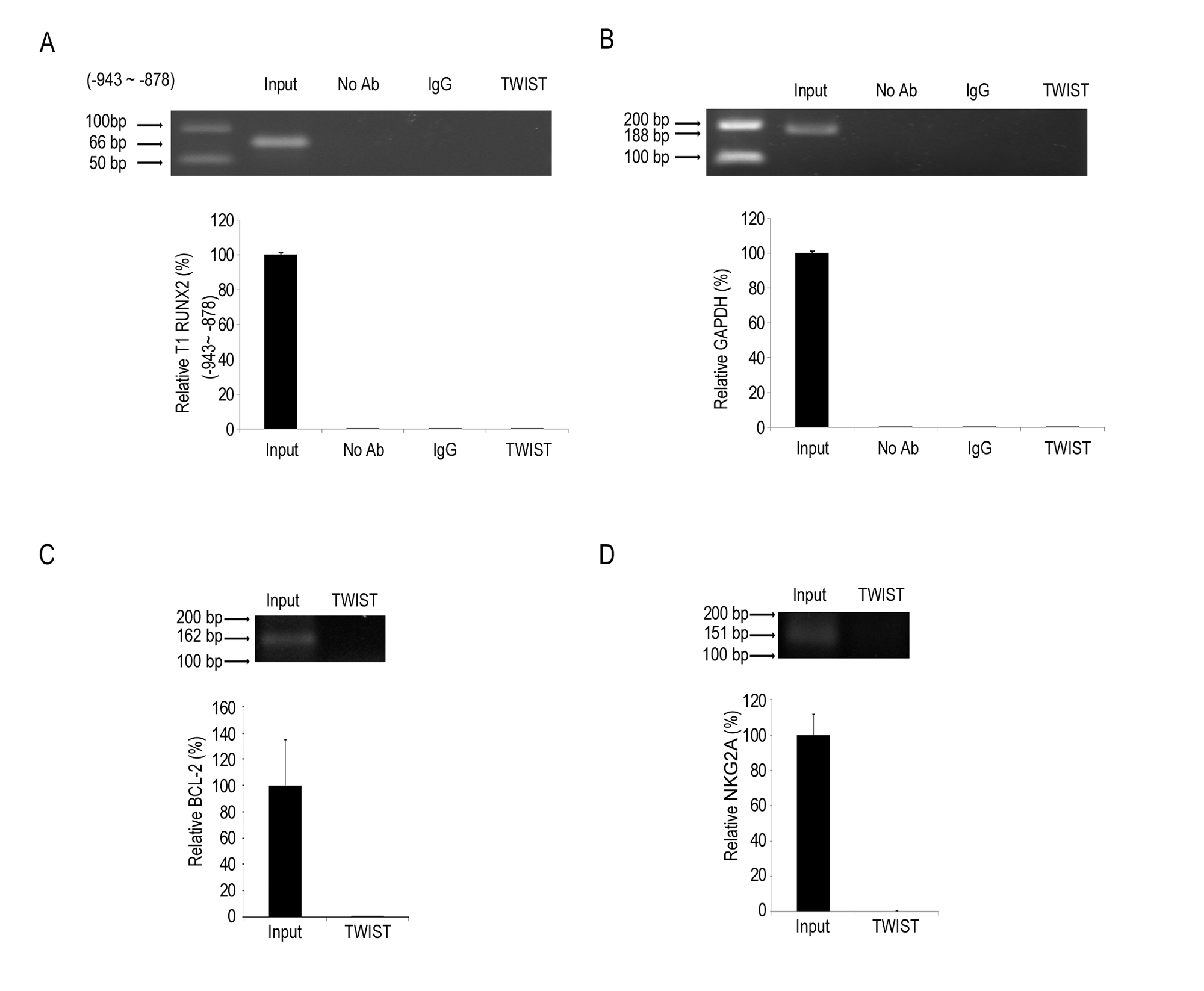

Supplement: Figure S4 — Unrelated fragments were used as internal control in ChIP analysis. ChIP analysis of MSCs after transfection with pFLAG-TWIST. The chromatin was incubated with anti-TWIST antibody. The internal control of T1RUNX2 promoter (−943 to −878) that does not contain the binding site (Figure S4A) and three unrelated promoters, GAPDH (Figure S4B), BCL-2 (Figure S4C) and NKG2A (Figure S4D) were amplified by PCR (upper panel) and quantified with quantitative RT-PCR (lower panel). Input, 2% of total input lysate. Results are shown as the mean ± SD values. (TIF) [file pone.0023965.s004.tif]
